# Bystander Phage Therapy: Inducing Host-Associated Bacteria to Produce Antimicrobial Toxins against the Pathogen Using Phages

**DOI:** 10.3390/antibiotics7040105

**Published:** 2018-12-04

**Authors:** T. Scott Brady, Christopher P. Fajardo, Bryan D. Merrill, Jared A. Hilton, Kiel A. Graves, Dennis L. Eggett, Sandra Hope

**Affiliations:** 1 Department of Microbiology and Molecular Biology, Brigham Young University, Provo, UT 84602, USA; thomasscottbrady@gmail.com (T.S.B.); christopher.fajardo@gmail.com (C.P.F.); brymerr921@gmail.com (B.D.M.); thehumanjervis@gmail.com (J.A.H.); kielgraves@gmail.com (K.A.G.); 2 Department of Statistics, Brigham Young University, Provo, UT 84602, USA; theegg@byu.edu

**Keywords:** American Foulbrood, bacteriophage, phage, phage therapy, *Paenibacillus larvae*, *Brevibacillus laterosporus*, treatment, safety, bystander phage therapy

## Abstract

*Brevibacillus laterosporus* is often present in beehives, including presence in hives infected with the causative agent of American Foulbrood (AFB), *Paenibacillus larvae*. In this work, 12 *B. laterosporus* bacteriophages induced bactericidal products in their host. Results demonstrate that *P. larvae* is susceptible to antimicrobials induced from field isolates of the bystander, *B. laterosporus*. Bystander antimicrobial activity was specific against the pathogen and not other bacterial species, indicating that the production was likely due to natural competition between the two bacteria. Three *B. laterosporus* phages were combined in a cocktail to treat AFB. Healthy hives treated with *B. laterosporus* phages experienced no difference in brood generation compared to control hives over 8 weeks. Phage presence in bee larvae after treatment rose to 60.8 ± 3.6% and dropped to 0 ± 0.8% after 72 h. In infected hives the recovery rate was 75% when treated, however AFB spores were not susceptible to the antimicrobials as evidenced by recurrence of AFB. We posit that the effectiveness of this treatment is due to the production of the bactericidal products of *B. laterosporus* when infected with phages resulting in bystander-killing of *P. larvae*. Bystander phage therapy may provide a new avenue for antibacterial production and treatment of disease.

## 1. Introduction

*Brevibacillus laterosporus* is a Gram-positive, spore-forming bacterium that can be found in myriad locations including the gut of honeybees [1,2,3,4,5]. While typically found at low levels in healthy honeybees, the population of *B. laterosporus* often increases as a secondary infection when a hive is infected with *Paenibacillus larvae* or *Melissococcus plutonius*, the causative agents of American Foulbrood and European foulbrood, respectively [6]. American Foulbrood (AFB) is the most devastating bacterial infection in honeybees, killing honeybee larvae and spreading easily from hive to hive within an apiary [7,8,9]. In the wake of antibiotic resistance in *P. larvae*, novel methods for controlling AFB outbreaks are needed, similar to the need for new approaches to treating antibiotic resistant bacterial infections in general.

Strains of *B. laterosporus* produce potent toxins that can kill a wide range of organisms [5,10,11]. *B. laterosporus* has been used as a bio control agent for decreasing the populations of unwanted bacteria and this method yielded modest results in attempts to control American Foulbrood [12,13]. While typically a symbiote to honeybees [14], *B. laterosporus* can produce toxins with insecticidal properties and certain strains of the bacterium are implicated in causing minor disease in honeybee hives after a primary infection [15,16,17,18]. The role of *B. laterosporus* as either a beneficial symbiote or as an opportunistic infector is yet to be fully understood.

Prior to this study, phages that specifically infect *B. laterosporus* were isolated from beehives and the genomes of most have been studied and published [19,20,21]. In this study, isolated phages were tested against strains of *B. laterosporus* to determine the most effective combination of phages to be included in a final cocktail. During isolation and experimentation, we discovered that when *B. laterosporus* was treated with phages, the bacteria began to produce antimicrobials that kill *P. larvae* when undiluted. These findings led us to believe that *B. laterosporus* phages could be used as a biocontrol for AFB by inducing antimicrobial production to kill *P. larvae*.

The studies presented here show: (1) The host range of identified phages; (2) the phages’ presence and persistence in the larval gut after treatment; (3) the phages’ ability to induce antimicrobial production compared to other forms of induction; (4) the phages’ safety to healthy honeybee hives over time; and (5) the phages’ ability to control an active AFB infection. We propose a new approach called “bystander phage therapy” as a method for treating pathogenic bacteria. 

## 2. Results

### 2.1. Phage Characteristics and Host Range

The genome sequences for all of the phages used in these studies, except for Lauren and Fawkes, were previously sequenced and analyzed [19,21]. GenBank accession numbers for the phage genomes are as follows: Jimmer1-KC595515, Jimmer2-KC595514, Emery-KC595516, Abouo-KC595517, Davies-KC595518, Osiris-KT151956, Powder-KT151958, SecTim467-KT151957, Sundance-KT151959, Jenst-KT151955.

**Figure 1:**
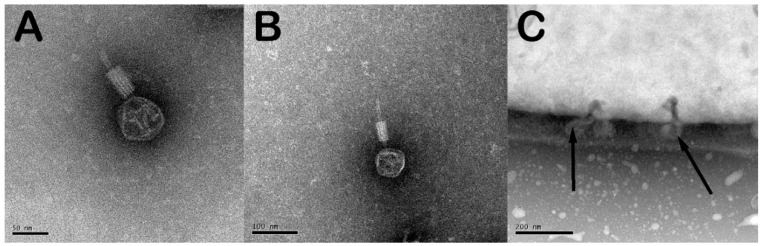
*Brevibacillus laterosporus* phages Lauren and Fawkes. (**A**) Single Lauren phage particle SEM image. (**B**) Single Fawkes phage particle SEM image. (**C**) Fawkes phage particles attached to BL2 bacterium SEM image, arrows point to attached phage particles. Images of the other phages mentioned were previously published by [19] and [21].

Electron microscopy images of Jimmer1, Jimmer2, Emery, Abouo, Davies, Osiris, and Powder were previously published [19,21]. Figure 1 includes electron microscopy images of the two previously unpublished images of phages used in this study, Lauren and Fawkes, from phage lysates. Figure 1C is an image of Fawkes attached to the side of BL2 *B. laterosporus* field isolate. 

Upon isolation, *B. laterosporus* phages were challenged for their ability to infect three field isolates of *B. laterosporus* as well as nine type-strains of *B. laterosporus* from the Bacillus Genetic Stock Center (BGSC) by both spot tests and plaque formation assays. Table 1 indicates bacterial susceptibility to *B. laterosporus* phage infection using *P. larvae* bacteria as a negative control. Emery/Abouo had the largest host range against archived *B. laterosporus* strains, showing infectivity against eight of the 12 strains. Fawkes showed infectivity against seven strains of which three were not covered by Emery/Abouo. None of the tested *B. laterosporus* phages were capable of forming plaques on lawns of 40A4. Furthermore, no plaques formed on *P. larvae* ATCC 9545, a highly phage susceptible strain [22], indicating that the isolated phages are specific to *B. laterosporus* and do not have the ability to cross-infect into *P. larvae*. 

### 2.2. Phage Persistence in the Larval Honeybee

**Figure 2:**
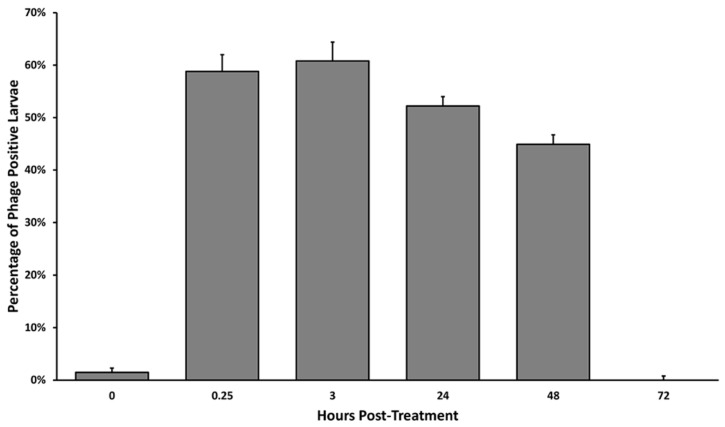
Average presence of phages in larvae samples after treatment. The time 0 sampling was taken just prior to initial treatment to serve as a baseline. Bees and racks were sprayed with phages and larvae were plucked from the racks at each timepoint and tested for the presence of phages.

This study aimed to determine whether phages would reach the larval gut and how long the phages would persist in a larval gut. Five hives were previously established in a single apiary and each hives’ brood racks (with the worker bees covering the brood) were sprayed with *B. laterosporus* phage lysate suspended in sugar water. One hundred larval specimens were collected from each hive at spaced time points and were tested for the presence of viable phages, see Figure 2. The first samples were collected at time 0 immediately prior to treatment with the phage cocktail to establish a baseline for the presence of naturally occurring phages in honeybee larvae. Phage persistence studies showed that phage presence in bee larvae was 1.5 ± 0.8% before treatment and rose to 58.8 ± 3.2% 15 min after treatment, 60.8 ± 3.6% after 3 hours, 52.2 ± 1.8% after 24 h, 44.9 ± 1.8% after 48 h, and 0 ± 0.8% after 72 h. Phages were found in larvae within 15 min of the treatment and peaked at 3 hours where 60.8 ± 3.6% of larvae contained detectible, viable phages as determined by spot test. Phage presence in bee larvae remained well above the normal untreated control for 2 days after the treatment was administered. After 3 days, the phage presence returned to the normal nominal levels.

### 2.3. Phage Infection Induces B. laterosporus to Produce Antimicrobials

**Figure 3:**
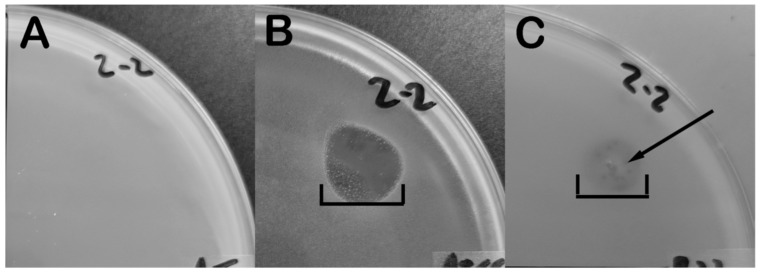
*B. laterosporus* antimicrobial product spot test. Drops of *B. laterosporus* phage lysate were placed and incubated for 24 h onto (**A**) a lawn of *A. tumefaciens* that did not respond to the antimicrobial product or generate plaque clearings, (**B**) a lawn of *P. larvae* that exhibited antimicrobial death, and (**C**) a lawn of *B. laterosporus* strain BL2 that showed antimicrobial death as well as phage infection formation. Brackets indicate antimicrobial clearing, arrow indicated phage plaque formation.

During culture of *B. laterosporus* phages, we observed that bacterial lawns exhibited clearing from phage plaques as well as a diffusion of a bacterial component in the vicinity of a plaque. An experiment was designed to characterize the effects of *B. laterosporus* phage on the production/release of toxins from *B. laterosporus*. Strains BL-2 and BL-6 were infected with the phages Fawkes and Emery/Abouo, respectively in duplicate. The resulting lysates were filtered and three µLs spotted onto lawns of different bacteria. Antimicrobial activity was qualified by the creation of a hole in the bacteria on the plate indicating cell die off distinguished between plaques from phages by observing the shape and size of the clearing (Figure 3, Table 2). Lysates from Fawkes and Emery/Abouo both contained antimicrobial products that were lethal to BL-2, BL-6, *P. larvae* ATCC 9545, and *E. coli* MG1655. Neither lysate type was effective against *Agrobacterium tumefaciens* or *Sinorhizobium meliloti*. 

These data indicate the sensitivity of *P. larvae* to the antimicrobial product generated by *B. laterosporus*, and that it has limited killing against other bacteria.

Control samples recreated various stages of the phage life cycle to verify phage-induced antimicrobial production as opposed to products release from other mechanisms. Supernatant from UV killed bacteria was spotted onto lawns of bacteria to identify if bacterial death alone induces antimicrobial production. The supernatant from mechanically lysed bacteria was also tested to determine whether phage lysis releases antimicrobial products present in the bacterial cytoplasm. Supernatant from untreated vegetative *B. laterosporus* was also tested to identify whether unprovoked bacteria releases antimicrobial products. None of the control sample supernatants formed holes in bacterial lawns, indicating that these mechanisms did not result in any production or release as seen in Figure 3. The lack of antimicrobial production via UV killing and mechanical lysis indicates that the bactericidal produced by *B. laterosporus* is not a result of bacterial death or lysis. Phage-induced antimicrobial production may be the result of expression of genes encoded by the bacteria since no known antimicrobial genes reside in the sequenced phage genomes while several have been identified in *B. laterosporus* [5,10]. The fact that more than one genetically unique *B. laterosporus* phage can induce the bacteria to make an identically-acting antimicrobial (Table 3 and [19]), may further suggest that the product arises from the bacterial genome instead of the phage genome.

### 2.4. Phage-Induced B. laterosporus Antimicrobial Products Shows Inert Characteristics Against Honeybees

This study aimed to determine whether phage treatment for *B. laterosporus* would be problematic for honeybees. Since *B. laterosporus* has been suggested previously to be a commensal to honeybees, this study was conducted to observe if side effects of phage-induced toxin or phage killing of *B. laterosporus* in the bee gut would decrease the overall health of the hives. Twelve hives, six in a test group and six in a mock-treated group, were installed into new boxes with new frames in spring. New queens and approximately 1.1 kg of honeybees were installed into each box and weekly inspections were made to follow the bees’ progress by observing the number of bees in the spaces between racks. Hives were allowed to become established for 9 weeks before receiving phage or mock treatments. Populations in all treated and untreated hives stayed below four full racks through early-summer. In mid-summer, the bees began to expand to fill the fourth rack, at which point the phage treatment commenced. 

**Figure 4:**
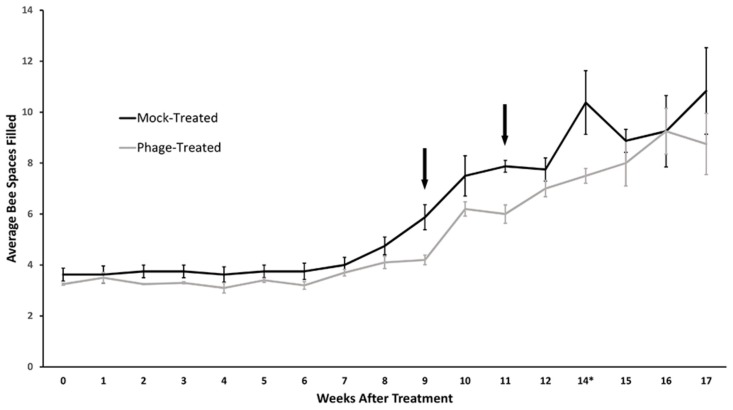
Colony expansion after phage treatment in beehives. New packets of bees with a fertilized queen were allowed to establish in new hives. Arrows indicate when phage treatments were administered to the bees and results demonstrate that healthy hives treated with *B. laterosporus* phage cocktail exhibited no difference in colony expansion when compared to healthy control hives. Bee spaces indicate honeybee population within the hive. Data not collected for week 13.

All 12 hives were treated three times at weeks nine and eleven, with 3 days between treatments for each regiment. Our data showed that all hives expanded at approximately the same rate during the study, see Figure 4. There was no statistical difference between the expansion of the bee populations in mock-treated controls versus the phage-treated group. The data was evaluated statistically using the repeated measures, mixed procedure, two-tailed analysis of the number of bee-filled spaces in the treated, and control hives over the 17-week period using an alpha level α = 0.05 (*p*-value of 0.1104). These data indicate that antimicrobial products in the phage lysate treatment was sufficiently low or not active on honeybees. Further, it shows that the hives were either lacking *B. laterosporus* and thus this bacterium is not essential for honeybee health, and/or that any antimicrobials or killing from phage infection of *B. laterosporus* does not adversely affect honeybee expansion. 

### 2.5. B. laterosporus Phages Can Effectively Treat an Active AFB Infection

The objective of this experiment was to determine the effectiveness *B. laterosporus* phages in curing honeybee hives of American Foulbrood caused by *P. larvae*. Forty hives of honeybees (*Apis mellifera*) were previously established in one apiary. Of the 40 colonies, 12 presented with American Foulbrood, the remaining 28 colonies were relocated to prevent the spread of the disease to the remaining healthy hives. Government regulation requires immediate treatment or destruction of known American Foulbrood-infected hives and any hives potentially exposed during an outbreak. Beekeepers are allowed to treat sick bees for 2 weeks at which point recovered hives can be kept and any hives with signs of the disease must be burned. Within these regulatory parameters, the 12 sick hives were all assigned to receive phage therapy and were followed for 2 weeks. All 12 sick hives were treated three times (each treatment was given 3 days apart) by spraying each rack on both sides with *B. laterosporus* phages in sugar water. The remaining 28 hives appeared healthy and were treated with antibiotics by the beekeeper. Treatment of the beehives occurred immediately before the onset of winter. 

All 40 hives were inspected 2 weeks after the first treatment. Of the 12 infected hives treated with *B. laterosporus* phages, 9 recovered and showed no signs of AFB upon inspection at week two, which indicates a 75% cure rate (see Table 3). Of the 28 originally uninfected hives, two appeared healthy at the beginning of the study but were diagnosed with AFB at the two-week inspection. The two hives had received antibiotic treatments along with the other 26 healthy hives; however, both hives collapsed with severe signs of AFB. It is anticipated that the hives were in very early stages of infection at the time of the outbreak and were missed as infected during hive assessment at the beginning of the study. The complete AFB destruction of these two untreated hives within 2 weeks of the first inspection of the study indicates that the 12 diagnosed-infected hives which received bystander phage therapy from that apiary during that outbreak were infected with a strain of *P. larvae* that was both antibiotic resistant and lethal. Dead-out hives were burned. No further problems were reported with the other 26 hives healthy antibiotic treated hives. From the 12 sick beehives, dead larval samples were taken from the hives before the first phage treatment and healthy larvae were taken at 2 weeks post-phage treatment (healthy larvae were taken post-treatment because no dead larvae were observed). The larval samples were analyzed by PCR for the presence of bacteria as described in the Materials and Methods Section 4.1. Results of PCR confirmed the presence of *P. larvae* and *B. laterosporus* DNA at pre-treatment, and only *B. laterosporus* DNA with no amplification of *P. larvae* DNA at post-treatment from the larval samples. The nine hives that recovered from AFB were followed through winter. In spring, of the nine recovered hives, five survived, four died. No signs of AFB were found in the four dead hives; three of the hives appeared to have frozen to death, and one hive was destroyed by vandals.

### 2.6. B. Laterosporus Phages Do not Prevent Reinfection by Latent P. larvae Spores

The five recovered, surviving hives were followed for 9 months to investigate the effectiveness of the *B. laterosporus* phage cocktail in the inactivation of latent *P. larvae* spores, see Table 4. Two weeks after phage treatment as well as in the following spring, 16 weeks after the first treatment, all five hives had no signs of AFB infection. At 18 weeks, one of the five surviving hives experienced an AFB infection and that hive recovered after another treatment of *B. laterosporus* phages. At each time of recurrence, all five hives in the apiary were preemptively treated with the phage cocktail. At week 22, the first hive and a second hive experienced symptoms of AFB, which were again treatable with *B. laterosporus*, signs of AFB disappearing within a week of the treatment. By week 26, all five hives presented with AFB symptoms and were treated with the phage cocktail, which again cleared all of the hives of AFB symptoms with 2 weeks. All hives were destroyed mid-summer due to the reoccurring infections. These data indicate that *B. laterosporus* phage treatment could kill active *P. larvae* infections, but could not kill *P. larvae* spores nor prevent future infection. 

## 3. Discussion

The phage cocktail used in these studies was formulated to specifically infect a wide range of *B. laterosporus* field isolate strains. As seen in [23] and [24], phage cocktails designed in this manner (with phages in the cocktail selected according to the ability to kill as many field strains as possible) are effective at reducing the amount of their target bacteria. Here, we observed that the phages selected for a cocktail using laboratory-generated data of phage efficacy was predictive of the efficacy of phages in field tests, as observed by the reduction in the amount of *P. larvae* DNA present in hives and recovery of 75% of the hives with 2 weeks of bystander phage treatment. Furthermore, we studied the antimicrobial-inducing capabilities in the laboratory to observe whether or not the antimicrobials could be effective at reducing *P. larvae* bacteria, and then applied our results to safety and efficacy studies in the field. 

Using *B. laterosporus* phages as a biocontrol comes with some inherent risk. We were concerned to know whether, by inducing antimicrobial synthesis and lysing *B. laterosporus*, the phage cocktail could release toxins with insecticidal properties or other adverse effects in honeybees. No such deleterious effects were seen in our studies. Firstly, we observed rapid loss of detectable phages in healthy larvae which indicates that a phage treatment has a relatively short exposure time to the bees. Secondly, we observed no short-term or long-term harm to healthy honeybees treated with multiple doses of phages. These studies add to the expanding literature that indicates that phage cocktails are a safe alternative to traditional antibiotic use [25,26,27,28,29,30,31]. 

The results of our studies further indicate that *B. laterosporus* is not a necessary symbiote for honeybee health, which conclusion is contrary to the postulations of several other researchers [11,12] but supports reports by others [5]. The current field of research surrounding *B. laterosporus* is tempestuous as to its merits and disadvantages. However, the research conducted in this article is uniquely equipped to demonstrate the effects of beehives with and without *B. laterosporus* in vivo and the results indicate that there are no significant differences between hives with or without the bacteria. This study also demonstrates advantages to having the bacteria naturally present and using phages to induce antimicrobials to kill pathogenic bacteria. 

**Figure 5:**
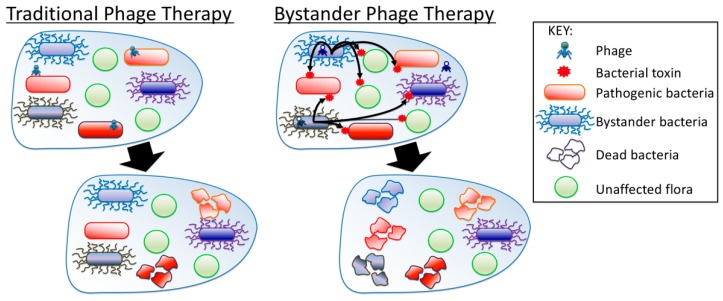
Mechanism of pathogen killing using phage therapy versus bystander phage therapy. In traditional phage therapy, phages against a pathogenic bacterium bind and lyse come bacterial strains, but may leave others unscathed (Left Panel). In Bystander Phage Therapy, phages against a bystander induce the bystander to make a toxin that kills all versions of the pathogenic bacteria while leaving an untouched population of itself that was not infected by phages (Right Panel).

One aim of our studies was to determine whether a phage cocktail designed for a co-infecting of commensal bacteria (*B. laterosporus*), could reduce the presence of a pathogenic bacteria (*P. larvae*), during a disease state (AFB infection). Figure 5 depicts this new “bystander phage therapy” as a phage treatment approach compared to the current dogma of phage therapy. Such situations may be more common than just this *B. laterosporus/P. larvae* system because co-existing, non-pathogenic bacteria may evolve to secrete antimicrobials in order to out-compete a pathogenic bacteria. The “bystander” bacteria may be poised to produce antimicrobials under stress as we were able to do using phage infection. It is important to note that none of our *B. laterosporus* phages could infect *P. larvae*. Therefore, any activity of a cocktail of *B. laterosporus* phages against AFB must be either from antimicrobial release to induce bystander killing of AFB or that *B. laterosporus* is responsible for AFB. We do not believe the latter is true. It was already known that *B. laterosporus* can produce antimicrobial toxins [10,32] and results from our laboratory experimentation demonstrate that these compounds are effective against *P. larvae* as well as other unrelated bacteria. In addition, we demonstrated that the phage cocktail can clear an active AFB infection but is not curative as observed by the recurrent infections. We hypothesize that the antimicrobials released by the phage when infecting *B. laterosporus* are effective against the vegetative bacteria that infect the larval brood, but that the antimicrobials are not able to eradicate *P. larvae* spores. *P. larvae* spores resistant to the antimicrobial toxins, unfortunately indicate that in this system bystander phage therapy is not a preventative tool for AFB but a treatment for clearing active infection, which is true of current antibiotic treatments for AFB which also leave *P. larvae* spores intact and viable. 

Furthermore, our results show that the antimicrobial from *B. laterosporus* was capable of killing vegetative *B. laterosporus*, albeit at a much lower sensitivity than killing of *P. larvae*. Typically, bacteria are good at defending themselves against their own agents while having potency against others. In this instance, we do see preference of the toxin against the pathogen but still some potential loss of the bystander. This discrepancy may be due to the fact that the toxin does not affect spores. *B. laterosporus* is also a spore-former, so its survival strategy may have evolved to release an antimicrobial that is effective against other bacteria at the risk of killing a few self-bacteria with the confidence that the majority of its own will survive as well as self-survival of its spores. This approach would explain the lack of toxicity to *P. larvae* spores as well. In application of bystander phage therapy to other bacterial commensal/pathogen systems, the lack of complete killing of the pathogen may not be an issue if the commensal bystander is not a spore-former or if the pathogenic bacteria is not a spore former.

Bystander phage therapy has an advantage over typical phage therapy because the range of targets affected by the antimicrobials can be much greater than traditional phage therapy that has limited host range. For instance, bystander phage therapy does not rely on the phage killing all of its targets. Rather, the phages only need to infect and induce enough antimicrobials to kill the pathogen. By this method, a hive could be infected with several strains of *P. larvae* that could include phage resistant *P. larvae* because of the limited host range of the individual phages, but the bacteria could still be killed by the phage-induced *B. laterosporus* antimicrobial product. This bystander effect could occur regardless of whether or not all strains of the non-pathogenic bacteria (*B. laterosporus*) are killed. An option not to kill all target bacterium is useful and desirable for a phage therapy approach because it means that the cocktail for bystander phage treatment would not need to include phages to kill every possible bacterial strain of its target. This simplifies the cocktail itself, and increases the chances of the treatment being functional since it is not dependent on killing all bystanders, but simply on activating the bystander to kill the pathogen. Bystander phage therapy may be a useful as an added component of phages in a traditional phage cocktail and/or in combination with antibiotics. Others have indicated the need for clinical phage treatments that include co-treatment of both phages and antibiotics at the time to restore antibiotic function as well as attempt complete elimination of all life-threatening bacteria [33]. Co-treatment of traditional phage and antibiotics may well indicate that bystander phages could step into the position of the antibiotic and a traditional/bystander cocktail of phages could be a highly effective therapeutic approach. 

Due to the nature of the antimicrobial effects of products made by *B. laterosporus*, bystander phage therapy could function as treatment against other bacterial infections in beehives such as *Melissococcus plutonius*, the causative agent of European Foulbrood. If the phage-induced antimicrobial products are lethal to other pathogens such as *M. plutonius*, then it would be an attractive alternative to standard phage therapies because of its ability to treat various diseases. This approach is especially helpful in the case of misdiagnoses of the pathogen causing foulbrood in a hive. For instance, *B. laterosporus* is often found in the hive regardless of whether foulbrood disease is due to the European or American Foulbrood pathogens (*M. plutonius* or *P. larvae,* respectively). Therefore, a bystander treatment using phages against *B. laterosporus* could be effective in both instances. Furthermore, *M. plutonius* is difficult and expensive to culture due to anaerobic requirements, which presents a barrier to lab work that would otherwise lead to phage isolation for traditional phage therapy against European Foulbrood. This exemplifies a situation where bystander phage therapy is a sensible method to pursue as a phage treatment since the bystander is easy to grow in aerobic conditions and could be used to treat more than one bacterial pathogen whose pathogenic presentation is very similar. Such approaches can be applied to many infectious bacterial systems. By inducing bystander bacteria to produce an antimicrobial, phages can remain a treatment option even for difficult-to-culture bacteria. 

## 4. Materials and Methods

### 4.1. Gathering B. laterosporus Field Isolates

Samples of honey and hive material were gathered from local apiaries and used for bacterial isolation. Samples were processed as described previously intended for *P. larvae* isolation [19,34] and isolated bacterial colonies were identified as *P. larvae* or *B. laterosporus* by PCR. Specifically, bacteria were initially streaked on *Paenibacillus larvae agar* PLA agar [35] and incubated at 37 °C. Catalase negative [36] and Gram-positive colonies were streaked on LB agar (Becton, Dickinson and Company, Sparks, MD, USA), gathered, archived in 20% glycerol, and stored at −80 °C. Bacteria were confirmed as *B. laterosporus* by PCR amplification of the *B. laterosporus rpo*B gene, see Table 5. Samples were also PCR tested with primers specific for *P. larvae* rpoB and *fts*A to confirm the presence of *P. larvae* [37]. Prior to PCR, bacterial samples were streaked out to single colonies. Template DNA for PCR was extracted by adding part of a colony to 50 μL of water in a PCR tube and incubating it at 100 °C for 10 min. The total PCR reaction volume was 25 μL composed of 22 μL standard PCR reagents (New England Biolabs, Ipswich, MA, USA) plus 3 μL of template DNA. After 30 cycles, PCR products were run in an agarose gel to confirm amplification. Amplicons from the reactions were sequenced using BigDye (Life Technologies, Carlsbad, CA, USA). MEGA6 was used to match sequence results with bacterial genomes. 

### 4.2. Isolating Phages Specific for B. laterosporus

*B. laterosporus* phages were isolated from bee debris collected near beehives. Bee debris was crushed and added to a flask containing LB broth and a field isolate of *B. laterosporus*. The bee debris and bacteria were incubated overnight at 37 °C. The mixture was spun in a centrifuge and the supernatant was passed through a 0.22 μm filter. A total of 50 μL of the supernatant were incubated at room temperature with 500 μL of *B. laterosporus* bacteria for 30 to 60 min, mixed with LB top agar, plated on LB agar, and incubated at 37 °C overnight. Plaques that appeared were isolated and re-plated a minimum of three times to purify individual phages. 

### 4.3. Host Range and Phage Presence Testing for Isolated Phages

*B. laterosporus* bacterial strains were tested for phage susceptibility using a plaque formation assay and a spot test assay. For the plaque formation assay, phage lysate was incubated at room temperature with 500 μL of an overnight culture of bacteria for 30 min, plated in 0.8% LB top agar, and incubated overnight at 37 °C. For the spot test assay, 500 μL of an overnight culture of bacteria was plated in 0.8% top agar. After the top agar hardened, 3 μL of phage lysate was placed on the top agar. The plates were incubated agar side facing up overnight at 37 °C. 

Phage detection in bee larvae was performed by taking one hundred larval samples at each time point and homogenizing them in 500 μL of LB broth in a 1.7 mL microcentrifuge tube for approximately 1 min. Three μL of the larval homogenate was spotted and incubated on plates *B. laterosporus* strains BL2 and BL6 were plated in top agar as described above.

### 4.4. Electron Microscopy

Phages were prepared for electron microscopy by incubating carbon-coated copper grids with 50 μL of high-titer lysate for 90 seconds, wicking away moisture, incubating with 50 μL of 2% phosphotungstic acid (pH = 7) for 90 seconds, wicking away moisture, and then allowing the grids to air dry prior to imaging. Electron micrographs were taken by the BYU Microscopy Center, and images were measured using ImageJ [42].

### 4.5. Creation of Bacterial Lysate to Test for B. Laterosporus and Phage Cocktail Treatments

Field isolates of *B. laterosporus,* BL-2 and BL-6, were reconstituted from freezer stock by plating onto Porcine Brain Heart Infusion (PBHI) (Acumedia, Lansing, MI) plates and incubating at 37 °C for 48 h. The resulting colonies were streaked for pure culture and incubated at 37 °C overnight. Fawkes and Emery/Abouo were brought out from freezer stock by streaking onto Porcine Brain-Heart Infusion (PBHI) plates with a lawn of *B. laterosporus* in agar incubated at 37 °C overnight. Picked plaques were grown in liquid culture with overnight growths of *B. laterosporus* to generate a high titer lysate. The lysates were centrifuged at 4000*g* for 30 min to pellet bacterial debris and then filtered (0.45 µm). The controls had no phage added and were processed the same to collect mock lysate.

Overnight cultures of *B. laterosporus* BL-2/BL-6, *P. larvae* ATCC 9545, *Agrobacterium tumefaciens* field isolate, *Sinorhizobium meliloti* field isolate, and *E. coli* MG1655 were plated using top agar onto plates of their respective media. Spot assays were conducted on bacterial lawns using three µL of lysate and incubating overnight. *A. tumefaciens* and *S. meliloti* samples were incubated at 30 °C and all other cultures were incubated at 37 °C.

Phages in the cocktail were generated as described above and then precipitated with polyethylene glycol (PEG) (Spectrum, New Brunswick, NJ) at 10,000*g* for 15 min at 4 °C to obtain a pure phage stock devoid of antimicrobial products. The cocktail was applied to the hives using a spray comprised of phage lysate diluted in a 1:1 sugar/water solution. Control hives received 340 mL of sugar water, while the phage treated hives received 320 mL of sugar water with 50 mL of phages containing a titer of 10^8^ mixed into the sugar water. 

### 4.6. Phage Beehive Parameters

In studies beginning with healthy hives, each had a viable laying queen, approximately 40,000 or more adult worker bees, uncapped brood, and no visible signs of American Foulbrood. Sick hives treated in Section 2.5 and Section 2.6 were identified by a local beekeeper and experimental treatment was approved through the Utah Department of Food and Agriculture. 

Population growth was determined in each of the hives based on the amount of racks the bees occupied. A rack was considered full when the space between the racks was fully crowded. In Section 2.4 the phage treatment started once all 12 of the hives achieved at least four fully occupied racks.

### 4.7. Statistics

The BYU statistical center analyzed the collected data to generate p-values, standard deviation, standard error, and to determine statistical significance. Statistical analysis included repeated measures, mixed procedure, two-tailed analysis using the Fisher’s exact test for 2 × 2 contingency tables with α = 0.05.

## 5. Conclusions

Phage therapies are an attractive alternative to traditional antibiotic use in the face of antibiotic resistance in pathogens. This study presents bystander phage therapy as a new alternative approach for phage therapy. The phages used in this study did not target the pathogen causing the disease that it treated, but rather targeted a known co-infecting bacterium and induced the co-infecting bacteria to produce antimicrobial products to which the pathogen is sensitive. 

The properties of phage-induced antimicrobials produced by *B. laterosporus* can be characterized to establish the extent of their host range. This research demonstrated that phages can induce *B. laterosporus* to produce antimicrobial products and demonstrated how phages that kill bystander bacteria can also result in killing of off-target, pathogenic bacteria. This approach could be useful as a single treatment for different diseases caused by different pathogens with overlapping symptoms provided that the phage-induced antimicrobial products can kill both pathogens, and that the loss of the antimicrobial-producing bystander bacteria is not vital to the organism. In this case, *B. laterosporus* is not a vital commensal and treatment of healthy bees with *B. laterosporus* phages did not result in any detectable health consequences in the bees. Use of *B. laterosporus* phages rescued a significant number of sick hives from succumbing to an antibiotic-resistant form of AFB. The use of bystander phage therapy is an exciting and new avenue of study that merits further investigation in the field of phage research.

## 6. Patents

System and Method for Treating a Disease or Bacterial Infection—Bystander Phage Therapy BYU#2018-037

## Figures and Tables

**Table 1:** Host range of *B. laterosporus* phages. Twelve *B. laterosporus* strains and one *P. larvae* strain were challenged with 12 *B. laterosporus* phages. The number of plus signs indicate the level of clearing. A minus sign indicates that no bacterial clearing occurred. BL2–BL14 are our field isolates of *B. laterosporus*, 40A1–40A10 are type strains of *B. laterosporus* from BGSC, and PL ATCC is the type strain of *P. larvae ATCC 9545*.

Phage	BL2	BL6	BL14	40A1	40A2	40A3	40A4	40A5	40A6	40A8	40A9	40A10	PL ATCC
Jimmer1	++++	−	++++	+	−	−	−	−	−	−	−	−	−
Jimmer2	++++	−	++++	+	−	−	−	−	−	−	−	−	−
Osiris	++++	−	++	++	+	+	−	+	++	−	++	+	−
Fawkes	++++	−	++	+++	+	−	−	−	+	−	++++	++	−
Lauren	++++	−	++++	+	−	−	−	−	+	−	+	+	−
Powder/ Sundance	+++	−	+++	+++	−	+	−	−	−	−	+++	−	−
SecTim467	+++	++	+++	++	+	−	−	−	−	−	+++	−	−
Jenst	−	++++	−	+	−	−	−	−	+	−	+++	−	−
Davies	−	++++	−	++++	++	+	−	+++	+++	+++	−	−	−
Emery/ Abouo	−	++++	−	++++	++++	+	−	+++	+++	++	+++	−	−

Underlines designate the bacteria used for phage isolation.

**Table 2:** Bacterial susceptibility to *B. laterosporus* antimicrobial products. *P. larvae, E. coli, A. tumefaciens, S. Meliloti,* and two strains of *B. laterosporus* were challenged with the supernatant from two phage lysates and the supernatant of live, dead, and mechanically lysed *B. laterosporus*. Antimicrobial-induced death is indicated by plus signs. A minus sign indicates no discernable antimicrobial clearing on the bacterial lawn.

Source Tested	B. Laterosporus (BL-2)	B. laterosporus (BL-6)	P. larvae	E. coli	A. tumefaciens	S. Meliloti
Emery/Abouo Phage lysate (BL-6)	+++	++ *	++++	+	−	−
Fawkes Phage lysate (BL-2)	++ *	+++	++++	+	−	−
Supernatant of live *B. Laterosporus*	−	−	−	−	−	−
Supernatant of UV killed *B. Laterosporus*	−	−	−	−	−	−
Supernatant of mechanically lysed *B. Laterosporus*	−	−	−	−	−	−

* Phage plaques were discernable on the bacterial lawns as well as death from antimicrobial products.

**Table 3:** Survival rate of hives after treatment in fall and after winter. Infected hives received phage cocktail treatments and uninfected hives were prophylactically treated with antibiotics. Survival rates of the hives were evaluated after 2 and 16 weeks.

Hive Status	Total Hives	AFB-Free Post-Treatment	Hive Survival Over Winter
Uninfected hives	28	92.85% *	78.1% ^†^
AFB infected hives	12	75%	62.5%

* Two hives of the 28 uninfected became infected when they were removed from the initial 12 infected hive. ^†^ Results excluding the two hives that became infected with AFB.

**Table 4:** Health of five hives after and AFB outbreak, *B. laterosporus* phage treatment, and overwintering. The surviving hives after *B. laterosporus* phage treatment were monitored for 28 weeks after initial treatment. When hives were seen to relapse, all hives were retreated with phage cocktail.

Hive Status	Week 16	Week 18 *	Week 20	Week 22 *	Week 24	Week 26 *	Week 28
Healthy	5	4	5	3	5	0	5
AFB+	0	1	0	2	0	5	0

* Weeks when phage cocktail was administered.

**Table 5:** Primer List. Primers used for amplification and sequencing of *rpo*B, *fts*A, and 16S rRNA genes of *B. laterosporus* and *P. larvae*. Results were used to positively identify bacterial isolates from beehives.

Primer	Sequence	Direction	Purpose	Reference
27F	5′-AGAGTTTGATCMTGGCTCAG-3′	Forward	16S rRNA universal primer	[38]
907R	5′-CCGTCAATTCMTTTRAGTTT-3′	Reverse		
BLrpoB-F	5′-GCAGGTAAACTGGTCCAGAGCG-3′	Forward	*B. laterosporus rpo*B	-
BLrpoB-R	5′-CACCTGTTGATTTATCAATCAGCG-3′	Reverse		
KAT1	5′-ACAAACACTGGACCCGATCTAC-3′	Forward	*P. larvae* ERIC-1 or ERIC-2	[39]
KAT2	5′-CCGCCTTCTTCATATCTCCC-3′	Reverse		
PLrpoB-F	5′-ATAACGCGAGACATTCCTAA-3′	Forward	Amplifies *P. larvae rpo*B	[40]
PLrpoB-R	5′-GAACGGCATATCTTCTTCAG-3′	Reverse		
PLftsA-F	5′-AAATCGGTGAGGAAGACATT-3′	Forward	Amplifies *P. larvae fts*A	[40]
PLftsA-R	5′-TGCCAATACGGTTTACTTTA-3′	Reverse		
ERIC1R	5′-ATGTAAGCTCCTGGGGATTCAC-3′	Forward	Generates multiple amplicons to fingerprint the bacteria tested	[41]
ERIC2	5′-AAGTAAGTGACTGGGGTGAGCG-3′	Reverse		
